# Global diastolic function in endurance athletes: three-dimensional volume tracking of the mitral annulus with cine-CMR

**DOI:** 10.1186/1532-429X-17-S1-Q6

**Published:** 2015-02-03

**Authors:** Vincent Wu, Sohae Chung, Myra S Cocker, Jacqueline Flewitt, Yoko Mikami, James A White, Matthias G Friedrich, Leon Axel

**Affiliations:** 1 Radiology, New York University School of Medicine, New York, NY, USA; 2 Libin Cardiovascular Institute of Alberta, University of Calgary, Calgary, AB, Canada; 3 Montreal Heart Institute, Montreal, QC, Canada; 4 University of Ottawa Heart Institute, Ottawa, ON, Canada

## Background

CMR can be used to assess the structure and function of athletes' hearts. However, diastolic function is not routinely measured with CMR. Mitral annular (MA) motion, commonly assessed by tissue Doppler imaging, is an important element of diastole that may help to distinguish physiological remodeling from pathological processes of the heart. CMR has been shown to play a useful role in evaluating MA motion. This study aims to characterize the global left ventricular diastolic function in endurance athletes by 3D volume tracking of the MA, using conventional cine-CMR images.

## Methods

CMR studies of 24 high-level endurance athletes (age 22 ± 3) were retrospectively selected from a database. Routine CMR studies from 39 patients with normal hearts (age 42 ± 16) and 29 hypertrophic cardiomyopathy (HCM) patients (age 52 ± 17) with normal ejection fractions were included for comparison. Using normalized cross-correlation, feature-tracking of the atrioventricular junctions was performed over the cardiac cycle, in routine long-axis 2-, 3-, and 4-chamber cine-CMR views. This resulted in 6 spatial MA points per cardiac phase, which were interpolated with a spline for 3D MA reconstruction. The 3D volume swept out by the MA was then calculated; the net sweep volume at a given cardiac phase was derived from the sum of the incremental volumes starting from end-diastole.

**Figure 1:**
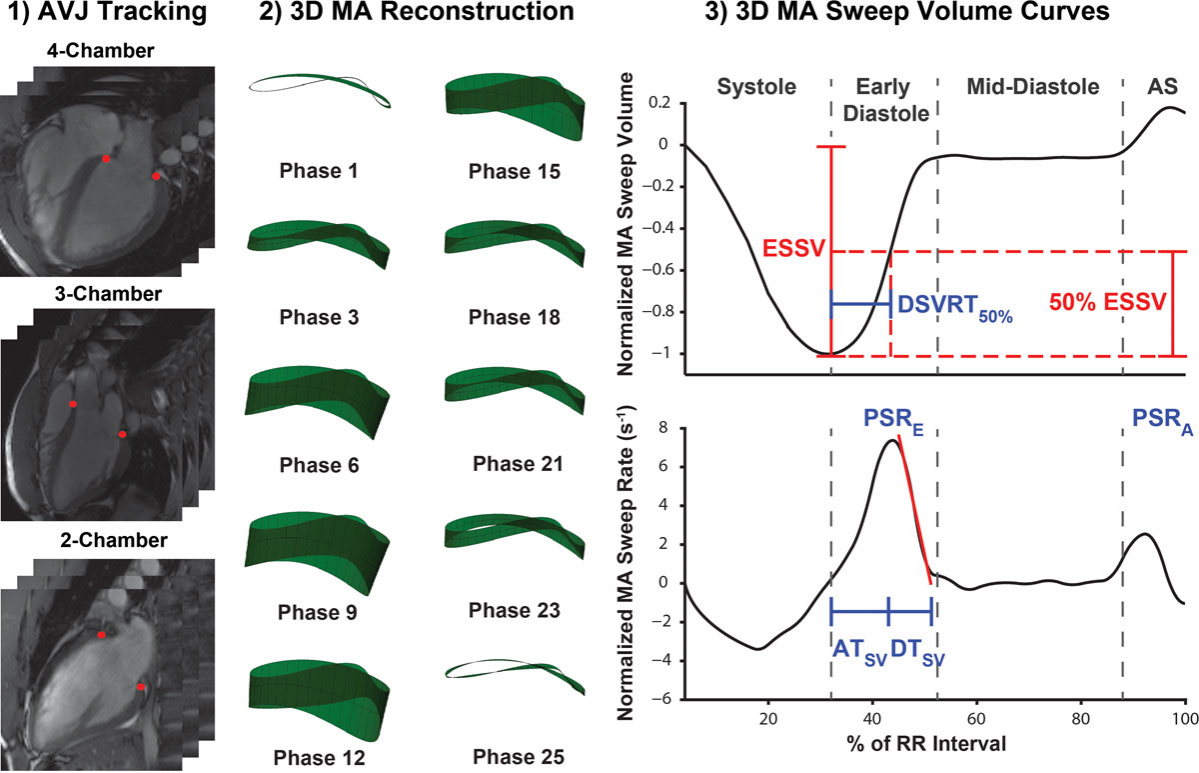
Feature-tracking of atrioventricular junctions (AVJ) in three long-axis cine-CMR image series over the cardiac cycle creates 6 MA coordinates per cardiac phase. These were used for 3D MA reconstruction and subsequent MA sweep volume calculations, producing CMR-derived diastolic parameters. ESSV = end systolic sweep volume; AS = atrial systole.

The resultant 3D MA sweep volume curve and its first derivative were calculated. The following diastolic variables were used (Figure 1): Peak sweep volume rates in early diastole (PSR_E_) and atrial systole (PSR_A_), normalized by end-systolic sweep volume (ESSV). Sweep volume acceleration time (AT_SV_) and deceleration time (DT_SV_) (relative to the PSR_E_ time). 50% diastolic sweep volume recovery (DSVRT_50_), defined as the time required in diastole for the MA to recover 50% of its ESSV, normalized by RR interval. Analysis of variance with post-hoc test and binary logistic regression were used for statistical analyses.

## Results

**Figure 2:**
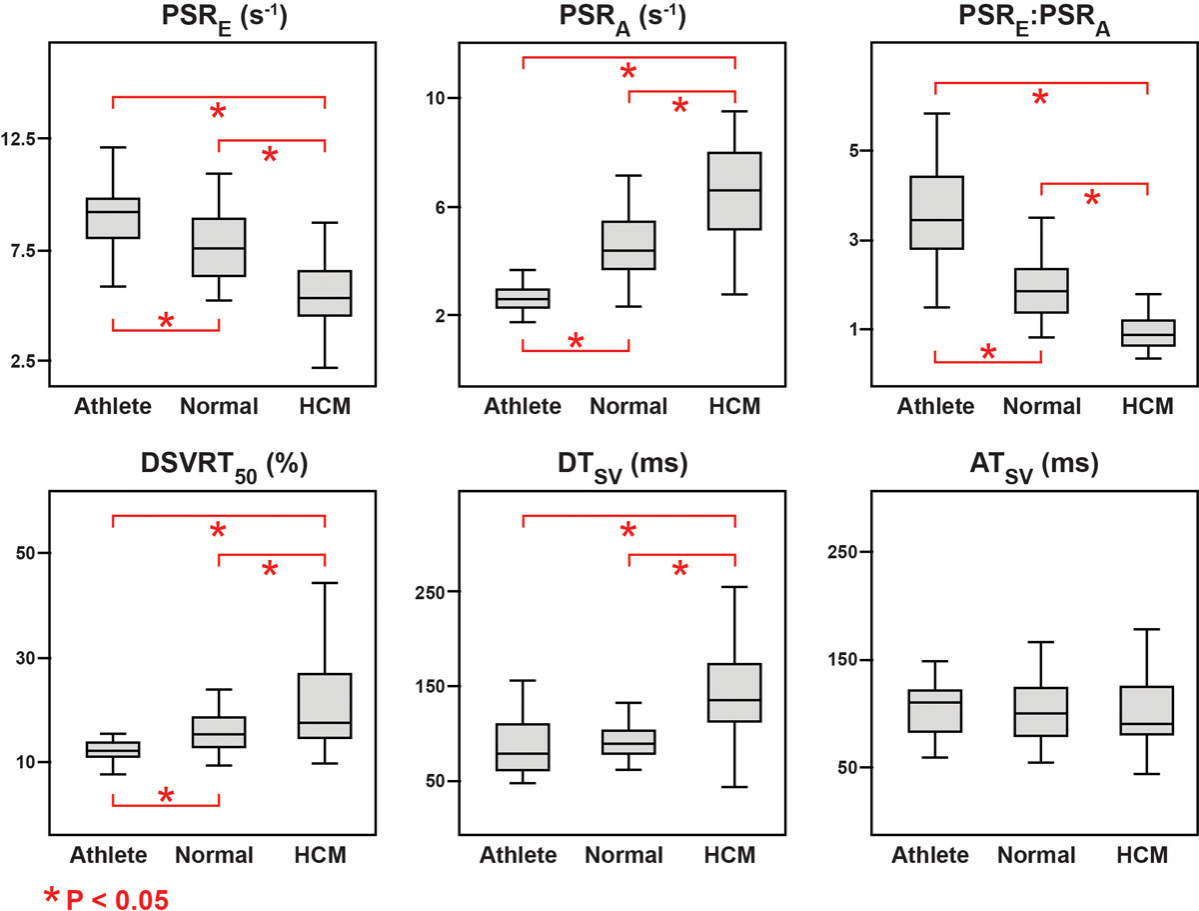
Comparisons of CMR-derived global diastolic function variables between the athletes, normal, and HCM groups.

As seen in Figure 2, athletes had the fastest PSR_E_, followed by the normal group and then the HCM group (9.0 ± 2.0 vs. 7.7 ± 1.6 vs. 5.7 ± 2.0 s^-1^, respectively, P < 0.05); PSR_A_ was the lowest in athletes, higher in the normal group, and the highest in the HCM group (2.7 ± 0.8 vs. 4.6 ± 1.3 vs. 6.4 ± 2.0 s^-1^, respectively, P < 0.05); DSVRT_50_ was the shortest in athletes, followed by the normal group and then the HCM group (11.9 ± 2.0 vs. 15.7 ± 4.1 vs. 22.1 ± 11.2 %, respectively, P < 0.05). No statistical differences were observed in AT_SV_ and DT_SV_ between athletes and the normal group. Lastly, the PSR_E_:PSR_A_ ratio was a significant independent predictor of athletes over the normal group when controlling for age (P = 0.035).

## Conclusions

3D MA sweep volumes derived from cine-CMR demonstrated that athletes had significantly faster early diastolic relaxation, followed by reduced MA motion in atrial systole, and faster diastolic MA sweep volume recovery, when compared to normal subjects and HCM patients.

## Funding

NIH R21-HL108218.

